# *PHACTR1* genetic variability is not critical in small vessel ischemic disease patients and PcomA recruitment in C57BL/6J mice

**DOI:** 10.1038/s41598-021-84919-x

**Published:** 2021-03-16

**Authors:** Clemens Messerschmidt, Marco Foddis, Sonja Blumenau, Susanne Müller, Kajetan Bentele, Manuel Holtgrewe, Celia Kun-Rodrigues, Isabel Alonso, Maria do Carmo Macario, Ana Sofia Morgadinho, Ana Graça Velon, Gustavo Santo, Isabel Santana, Saana Mönkäre, Liina Kuuluvainen, Johanna Schleutker, Minna Pöyhönen, Liisa Myllykangas, Assunta Senatore, Daniel Berchtold, Katarzyna Winek, Andreas Meisel, Aleksandra Pavlovic, Vladimir Kostic, Valerija Dobricic, Ebba Lohmann, Hasmet Hanagasi, Gamze Guven, Basar Bilgic, Jose Bras, Rita Guerreiro, Dieter Beule, Ulrich Dirnagl, Celeste Sassi

**Affiliations:** 1 Department of Experimental Neurology, Center for Stroke Research Berlin (CSB), Charité-Universitätsmedizin Berlin, Corporate Member of Freie Universität Berlin, Humboldt-Universität Zu Berlin, and Berlin Institute of Health, Charitéplatz 1, 10117 Berlin, Germany; 2 Berlin Institute of Health, BIH, Core Unit Bioinformatics and Charité-Universitätsmedizin Berlin, Berlin, Germany; 3 Center for Neurodegenerative Science, Van Andel Research Institute, Grand Rapids, MI USA; 4 CGPP and UnIGENe, Instituto Biologia Molecular Celular, Instituto de Investigação e Inovação em Saúde, Porto, Portugal; 5 Department of Neurology, Centro Hospitalar e Universitário de Coimbra, Coimbra, Portugal; 6 Department of Neurology, Centro Hospitalar Trás-os-Montes e Alto Douro, Vila Real, Portugal; 7 Faculdade de Medicina da Universidade de Coimbra, Coimbra, Portugal; 8 Centro de Neurociências e Biologia Celular da Universidade de Coimbra, Coimbra, Portugal; 9 Department of Medical Genetics, University of Helsinki, Helsinki, Finland; 10 Laboratory Division, Genomics, Department of Medical Genetics, Turku University Hospital, Turku, Finland; 11 Department of Clinical Genetics, Helsinki University Hospital, Helsinki, Finland; 12 Department of Pathology, University of Helsinki and Helsinki University Hospital, Helsinki, Finland; 13 Institute of Neuropathology, University of Zurich, Zurich, Switzerland; 14 Clinic of Neurology, Faculty of Medicine, University of Belgrade, Belgrade, Serbia; 15 Lübeck Interdisciplinary Platform for Genome Analytics (LIGA), Institutes of Neurogenetics & Cardiogenetics, University of Lübeck, Lübeck, Germany; 16 Behavioural Neurology and Movement Disorders Unit, Department of Neurology, Istanbul Faculty of Medicine, Istanbul University, Istanbul, Turkey; 17 Department of Neurodegenerative Diseases, Hertie Institute for Clinical Brain Research, University of Tübingen, Tübingen, Germany; 18 DZNE, German Center for Neurodegenerative Diseases, Tübingen, Germany; 19 Department of Genetics, Aziz Sancar Institute of Experimental Medicine, Istanbul University, Istanbul, Turkey; 20 Klinik und Poliklinik für Neurologie, Abteilung für Experimentelle Neurologie, Charité-Universitätsmedizin Berlin, Charitéplatz 1, 10117 Berlin, Germany

**Keywords:** Genetics, Clinical genetics, Disease genetics

## Abstract

Recently, several genome-wide association studies identified *PHACTR1* as key locus for five diverse vascular disorders: coronary artery disease, migraine, fibromuscular dysplasia, cervical artery dissection and hypertension. Although these represent significant risk factors or comorbidities for ischemic stroke, *PHACTR1* role in brain small vessel ischemic disease and ischemic stroke most important survival mechanism, such as the recruitment of brain collateral arteries like posterior communicating arteries (PcomAs), remains unknown. Therefore, we applied exome and genome sequencing in a multi-ethnic cohort of 180 early-onset independent familial and apparently sporadic brain small vessel ischemic disease and CADASIL-like Caucasian patients from US, Portugal, Finland, Serbia and Turkey and in 2 C57BL/6J stroke mouse models (bilateral common carotid artery stenosis [BCCAS] and middle cerebral artery occlusion [MCAO]), characterized by different degrees of PcomAs patency. We report 3 very rare coding variants in the small vessel ischemic disease-CADASIL-like cohort (p.Glu198Gln, p.Arg204Gly, p.Val251Leu) and a stop-gain mutation (p.Gln273*) in one MCAO mouse. These coding variants do not cluster in PHACTR1 known pathogenic domains and are not likely to play a critical role in small vessel ischemic disease or brain collateral circulation. We also exclude the possibility that copy number variants (CNVs) or a variant enrichment in *Phactr1* may be associated with PcomA recruitment in BCCAS mice or linked to diverse vascular traits (cerebral blood flow pre-surgery, PcomA size, leptomeningeal microcollateral length and junction density during brain hypoperfusion) in C57BL/6J mice, respectively. Genetic variability in *PHACTR1* is not likely to be a common susceptibility factor influencing small vessel ischemic disease in patients and PcomA recruitment in C57BL/6J mice. Nonetheless, rare variants in PHACTR1 RPEL domains may influence the stroke outcome and are worth investigating in a larger cohort of small vessel ischemic disease patients, different ischemic stroke subtypes and with functional studies.

## Introduction

Recently, genome-wide association studies (GWASs) identified Phosphatase and actin regulator 1 (*PHACTR1*) as a critical locus significantly associated to five different vascular disorders: coronary artery disease, migraine, fibromuscular dysplasia, cervical artery dissection and hypertension ^1–4^, which represent frequent and significant comorbidities or risk factors linked to ischemic stroke, particularly with early-onset ^5–10^. However, the potential pathogenic link between *PHACTR1* genetic variability and other cerebrovascular disorders leading to ischemic stroke or critically influencing its outcome, such as small vessel ischemic disease (SVID) and brain collateral artery plasticity like posterior communicating arteries (PcomAs) recruitment, have not been investigated. Interestingly, a recent study reported 2 de novo* PHACTR1* rare coding variants, mapping to the RPEL domains as causative factors for paediatric epileptic syndromes such as West syndrome and other neurodevelopmental disorders ^11^. Therefore, suggesting that the PHACTR1 locus may harbour rare coding disease-modifying variant(s), that are likely to remain undetected in GWASs either because they are not targeted in the GWAS array or because, even when applying genotype imputation, the detection of very rare coding variants remains inaccurate.

To overcome this drawback, in the last 10 years, deep resequencing studies have been powerful strategies to effectively complement GWASs and unveil rare coding functional variants in the GWAS susceptibility loci ^12–15^.

Finally, despite the critical importance of brain collateral arteries and, among these, PcomAs, their study in patients remains a major challenge, mostly due to their recruitment exclusively under moderate-severe acute hypoxic-ischemic conditions and their complex phenotype influenced by several additive factors (genetics, sex and aging) or comorbidities (diabetes and hypertension) and the absence of standardized methods for their accurate study ^16–18^.

By contrast, C57BL/6J inbred mice, minimizing genetic and environmental variability, offer an ideal system to study PcomA plasticity, whose main differences have been already characterized ^19–21^. Moreover, genome sequencing in different mouse strains shed light on the extensive similarities linking mouse and human genome and validated the importance of using mouse models to investigate the genetics of human diseases ^22,23^.

**Figure 1:**
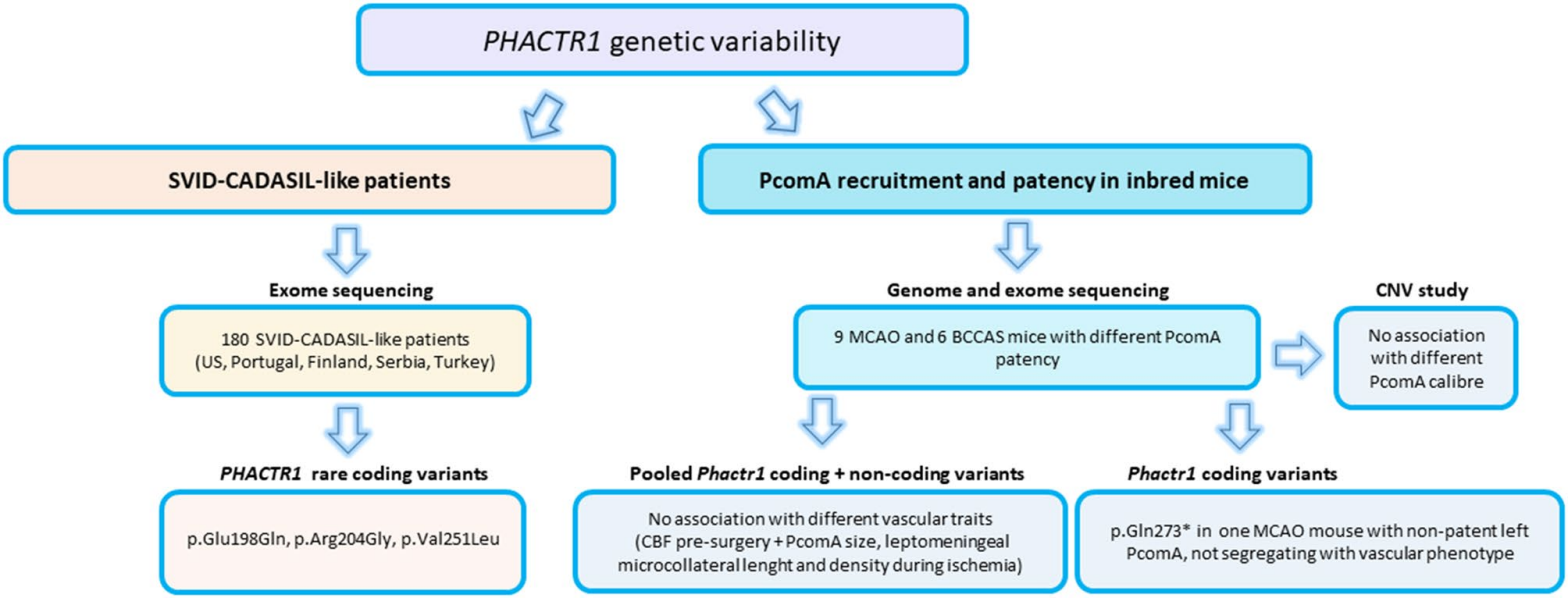
Pipeline followed in the present study. *SVID* small vessel ischemic disease, *US* United States, *BCCAS* bilateral common carotid artery stenosis, *ECA* external carotid artery, *PcomAs* posterior communicating arteries, *CNV* copy number variant, *MCAO* middle cerebral artery occlusion.

Therefore, to study the possible role of *PHACTR1* genetic and mostly rare coding variability in SVID-CADASIL-like patients and PcomAs recruitment during acute and subacute ischemia in C57BL/6J mice, we performed exome sequencing in a multi-ethnic cohort of 180 early-onset independent familial and apparently sporadic SVID and CADASIL-like Caucasian patients from US, Portugal, Finland, Serbia and Turkey and used a combination of complementary techniques (genome and exome sequencing, T2 weighted magnetic resonance imaging [T2-MRI], arterial spin labelling cerebral blood flow [CBF], magnetic resonance angiography [MRA] and histology) in two experimental mouse models of cerebral ischemia (bilateral common carotid artery stenosis [BCCAS] and middle cerebral artery occlusion [MCAO]) (Fig. 1). The results from this approach do not support a critical role of *PHACTR1* rare coding variants in SVID or CADASIL-like patients and PcomA patency during hypoxia–ischemia in mice.

## Materials and methods

### Patient cohort

The cohort was composed of 180 independent familial and early-onset apparently sporadic SVID and CADASIL-like Caucasian patients (96 and 84, respectively). Inclusion criteria for the SVID cohort included small vessel ischemic disease diagnosis based on TOAST classification, early age at onset (< 65 years [only two cases, whose age-at onset was 68 and 71 years old have been included in the study because familial and therefore enriched for genetic causative or risk factors]), absence of known pathogenic mutations in Mendelian small vessel disease genes (*HTRA1*, *NOTCH3*, *ACTA2* and *COL4A1*) and no enrichment for vascular risk factors except for hypertension, which generally plays a critical role in elderly people ^24^. The collection of samples included in this study has been approved by the ethics committee of the Faculty of Medicine, University of Coimbra, Coimbra, Portugal; the Ethics Committee of the School of Medicine, University of Belgrade (Serbia); the Ethics Committee of Istanbul Faculty of Medicine, Istanbul University and the Ethics Committee of the Hospital District of Southwest Finland. All NINDS Repository samples were collected only after an IRB-approved, signed informed consent was secured by the submitter. The diagnostic criteria for the CADASIL-like disease cohort were met by combining clinical symptoms, imaging data, and positive medical history in the absence of known *NOTCH3* pathogenic mutations and based on the previous literature ^25^. Ninety-six patients (53.3%) were from the US (NINDS Repository), 34 (18.9%) from Portugal, 33 (18.3%) from Finland, 15 (8.3%) from Serbia and 2 (1.1%) from Turkey. The mean age at disease onset was 52 years (SD = 10.9) and 76 (42.2%) had a positive family history. Among the comorbidities and possible risk factors, hypertension was reported in 44.4% of the patients, diabetes type 2 in 18.3%, cardio-vascular comorbidities (myocardial infarction, atrial fibrillation) in 12.2%, migraine in 10.0% and hypercholesterolemia in 7.2%. Given the prevalent role of hypertension and diabetes mellitus 2 in SVID in the elderly people ^24^ and the young age at onset of the cohort, these patients were considered enriched for genetic risk factors (Table 1). Finally, 478 controls > 60 years of age were selected from ‘HEALTHY EXOMES’, HEX, a publicly available database, which collects exome sequencing data from elderly neuropathologically proven controls (https://www.alzforum.org/exomes/hex) ^26^.

**Table 1:** SVID and CADASIL-like exome sequencing cohort.

Country of origin	N	Disease	AAO (SD)	M:F	N cases with family history (%)	Hypertension (%)	Diabetes (%)	N cases with migraine (%)	N cases with heart comorbidities (MI, AF) (%)	N cases with hypercholesterolemia (%)
US	96	SVID	51.5 (8.1)	0.82	43 (44.7)	58 (60.4)	29 (30.2)	NA	11 (11.4)	2 (2)
Portugal	34	CADASIL-like	44.5 (12.3)	0.36	9 (26.5)	NA	NA	9 (26.5)	1 (3)	NA
Finland	33	CADASIL-like	60.5 (12.2)	0.74	16 (48.5)	22 (66.6)	4 (12.1)	9 (27.3)	10 (30.3)	11 (33.3)
Serbia	15	CADASIL-like	NA	0.63	7 (46.6)	NA	NA	NA	NA	NA
Turkey	2	CADASIL-like	NA	0.5	1 (50)	NA	NA	NA	NA	NA
Total or average	180 (96 + 84)		52 (10.9)	0.71	76 (42.2)	80 (44.4)	33 (18.3)	18 (10)	22 (12.2)	13 (7.2)

*SVID* small vessel ischemic disease, *AAO* age at onset, *SD* standard deviation, *M* male, *F* female, *MI* myocardial infarction, *AF* atrial fibrillation.

### BCCAS and MCAO mouse model, experimental design and exclusion criteria

**Figure 2:**
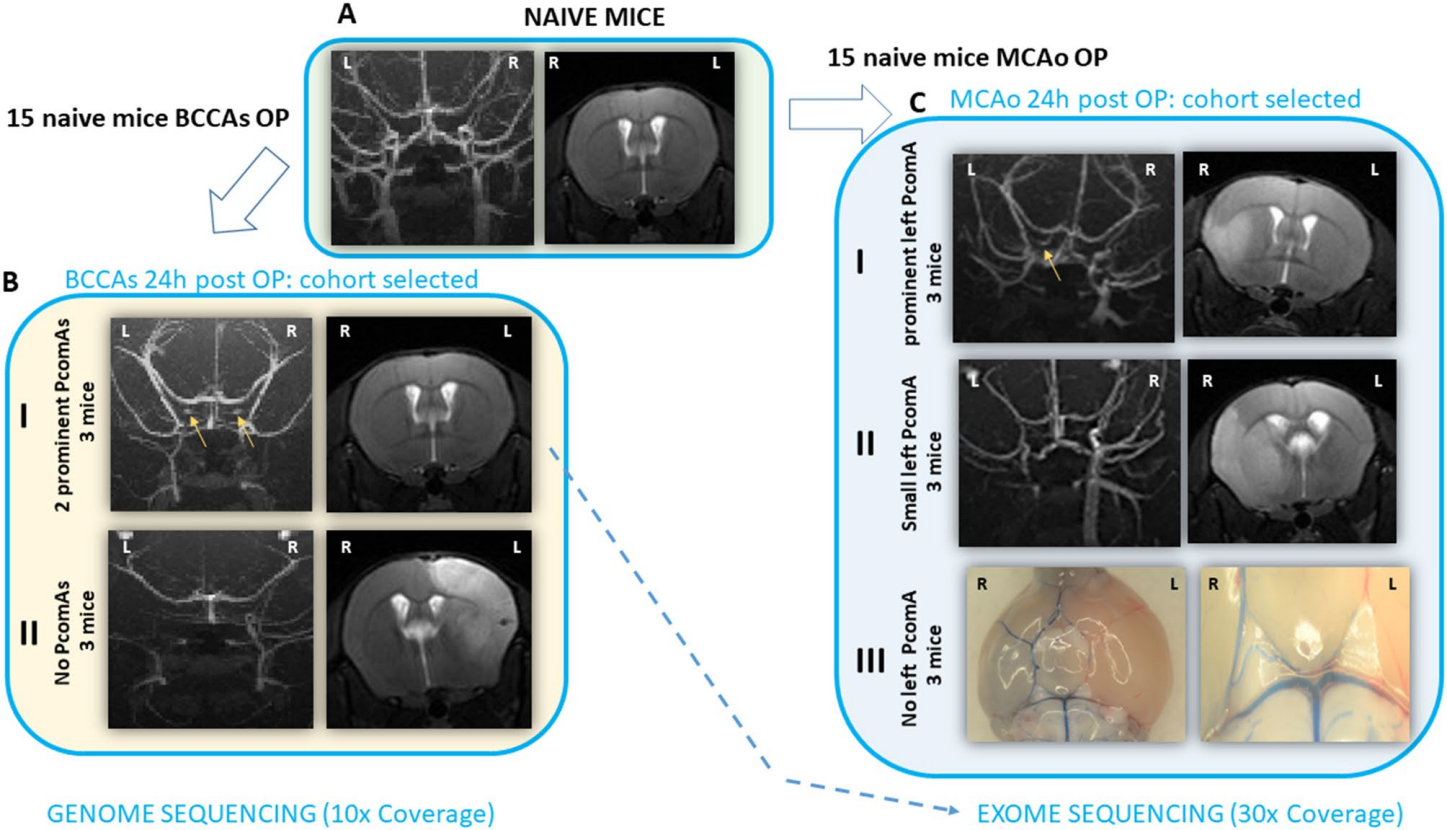
Pipeline used for the mouse cohort selection. Neuroimaging and vascular phenotypes of BCCAS and MCAO mice included in the study. Based on our previous studies with BCCAS and MCAO mice, we selected 2 cohorts of naive mice that underwent either the BCCAS or MCAO surgery (**A**). We then selected mice with extreme phenotypes in terms of PcomA patency based on the MRA, MRI and CBF measurement 24 h post surgery or, if not available (BCCAS mice with no PcomAs, dying a few hours post-surgery), based on histology. As expected and in line with our previous experiments we obtained 15% of mice with prominent PcomA(s), 25% of mice with non patent PcomA and the majority (60%) with small PcomA. Among these we then selected mice with extreme and dychotomic vascular phenotype. (**BI**) BCCAS mouse with two very prominent PcomAs (yellow arrows) and no ischemic lesions detectable on T2-weighted MRI. (**BII**) BCCAS mouse with no PcomAs, which developed a severe right hemispheric stroke and died 24 h after the surgery. (**CI**) MCAO mouse with left prominent PcomA (yellow arrow), which developed a small ischemic lesion (≈ 5% of the left hemisphere at 1 day) mostly affecting ventral areas (prefrontal cortex, striatum and ventral hippocampus). (**CII**) MCAO mouse with left small PcomA non detectable on MRA post-surgery, which developed a severe ischemic lesion (≈ 34% of the left hemisphere at 1 day) affecting also dorsal areas (orbital cortex and cerebellum). (**CIII**) MCAO mice with non-patent PcomA, which died between few hours and 2 days post surgery. *R* right, *L* left.

Experiments were approved by the Landesamt für Gesundheit und Soziales and conducted according to the German Animal Welfare Act and institutional guidelines. Thirty male C57BL/6J mice (purchased at 8 weeks of age, Charles River, Germany and 10 weeks of age Janvier France, respectively) were housed in a temperature (22 ± 2 °C), humidity (55 ± 10%), and light (12/12-h light/dark cycle) controlled environment. The animals underwent hypoperfusion between 9 and 13 weeks of age. Hypoperfusion was achieved by bilateral common carotid artery stenosis (BCCAS) (15 mice) or through 60 min transient blockage of left middle cerebral artery (MCA) and permanent occlusion of common carotid artery and external carotid artery (MCAO) (15 mice). Among these, 6 BCCAS and 9 MCAO mice with extreme phenotype were selected for the genetic study based on MRA, T2-MRI and CBF data 24 h post-surgery (Fig. 2).

The study was carried out in compliance with the ARRIVE guidelines.

### Mouse cohort for the genetic screening

Our mouse cohort was composed of six BCCAS and nine MCAO mice with different PcomA patency phenotypes (Table 2, Fig. 2). Selection of the mice was based on our previous and highly reproducible experiments in BCCAS and MCAO mice aimed at characterizing their vascular phenotype (^27^ and unpublished data). Inclusion criteria for this study were based on the brain acute hypoperfusion phase and particularly on MRA, T2-MRI scans and brain perfusion 24 h post-surgery (Fig. 2). The mouse phenotype was followed up and further characterized with additional MRI and MRA scans and histology at 1 week, 4 and 7 weeks.

**Table 2:** Mouse exome/genome sequencing cohort.

Mouse strain	Mouse model	Vascular phenotype^a^	Gender	Age	N	Sequencing strategy
C57BL/6J	BCCAS	2 very prominent PcomAs	M	10–12w	3	WGS WES
C57BL/6J	BCCAS	No prominent PcomAs	M	10–12w	3	WGS
C57BL/6J	MCAO	Left prominent/very prominent PcomA	M	10–12w	3	WES
C57BL/6J	MCAO	Left small PcomA	M	10–12w	3	WES
C57BL/6J	MCAO	Left non-patent PcomA	M	10–12w	3	WES

*PcomA* posterior communicating artery, *ECA* external carotid artery, *M* male, *w* weeks, *WGS* whole genome sequencing, *WES* whole exome sequencing.

^a^The vascular phenotype classification has been already described (Foddis et al.^27^).

The study of the PcomA role during acute hypoperfusion followed Martin et al. PcomA classification ^28,29^ and has been already described ^27^. Briefly, this identifies 4 PcomA classes, based on the ratio between PcomA and basilar artery (BA) diameter: (1) PcomA < 10% of BA; (2) PcomA 11–20% of BA; (3) PcomA 21–30% of BA and (4) PcomA > 30% of BA. We identify class 1 and class 2 as ‘non-patent’, class 3 as ‘small’, class 4 as ‘prominent’ and included a fifth class, represented by PcomA > 60% of BA, described as ‘very prominent’.

The diameters of the PcomAs were measured at the smallest point and the diameter of the BA was measured proximal to the superior cerebellar arteries both for the Evans Blue and fluorescent WGA stainings (MCAO mice) or only with for Evans Blue staining (BCCAS mice) with ImageJ. The diameter of the PcomAs as a percentage of the diameter of the BA was calculated and used in the analysis as previously described ^28,29^.

In our mouse cohort, BCCAS mice with extreme dichotomous vascular phenotype were selected for genome sequencing: three BCCAS mice with two very prominent PcomAs (Fig. 2-BI) and 3 BCCAS mice with no patent PcomAs, which died between few hours and one day after surgery due to very severe hemispheric strokes (Fig. 2-BII). Three of these with the very effective vascular phenotype have been included in the exome sequencing cohort, together with nine MCAO mice, characterized by different left PcomA calibre: (a) three MCAO mice with prominent-very prominent left PcomA, that developed small ischemic lesions (≈ 5–10% of the left hemisphere), mostly affecting ventral areas (prefrontal cortex, striatum and ventral hippocampus), and were characterized by the most favourable stroke outcomes (Fig. 2-CI); (b) three MCAO mice with small PcomA, that survived the surgery but developed monolateral severe left strokes affecting up to 34% of the left hemisphere and including also dorsal areas (orbital cortex and cerebellum) (Fig. 2-CII) and (c) three MCAO mice with non-patent PcomA, which died between few hours and 2 days post surgery (Fig. 2-CIII) (Table 2). Given the extreme inbreeding of the C57BL/6J strain, carefully inbred for over 70 years through more than 200 generations of brother-sister mating ^30^, and the likely minimal influence of environmental factors, these mice were genetically considered as members of the same large multigenerational family coming from a small and isolated village. Moreover, the selection of extreme phenotypes (absent-small PcomA vs prominent-very prominent PcomA), allowed us to reach an high power for the detection of rare variants with large effect size, despite the small sample size ^31,32^.

The BCCAS and MCAO mouse models are described in detail in the “Supplementary materials and methods”.

### Exome sequencing and genome sequencing in patients and mice

We performed whole exome sequencing (WES) on a cohort of 180 independent familial and early-onset sporadic SVID and CADASIL-like cases and in 12 C57BL/6J mice (nine MCAO and three BCCAS) and whole genome sequencing (WGS) in six C57BL/6J BCCAS mice. DNA was extracted from blood (patients) or cerebellum (mice) using standard protocols. Library preparation for next generation sequencing used 50 ng DNA. Exome libraries were prepared using Nextera Rapid Capture Exome and Kit (4 rxn × 12 plex, FC-140-1002) and Nextera DNA Library Prep Kit (FC-121-1030). The DNA library was then hybridized to an exome capture library (Nextera, Illumina Inc.) and precipitated using streptavidin-coated magnetic beads (Nextera, Illumina). Exome-enriched libraries were PCR-amplified, and then DNA hybridized to paired-end flow cells using a cBot (Illumina, Inc.) cluster generation system.

The WES libraries were sequenced paired-end 75 bp on Illumina HiSeq 4000 with a median of 60.5 million reads per library. The WGS libraries were sequenced paired-end 150 bp on Illumina NextSeq 500 with a median of 97.7 million reads per library.

### BCCAS and MCAO mouse model, histology

As previously described ^27^, PFA perfused brains were cut into 50-µm-thick sections on a cryostat. After washing with phosphate-buffered saline (PBS), free-floating sections were incubated with 10% normal goat serum (NGS, GeneTech, GTX27481) and 0.1% Triton-X-100 (Sigma-Aldrich, X100) in PBS for 1 h at room temperature to block unspecific binding. Primary and secondary antibodies were diluted in 1% NGS and 0.1% Triton-X-100 in PBS. Sections were incubated with rat anti-GFAP primary antibody (Millipore, 345860) for astrocytes and rabbit anti-PHACTR1 primary antibody (Invitrogen, PAS-44332) at 4 °C overnight. After thorough washing, sections were incubated at room temperature with AlexaFluor-594-conjugated goat anti-rat (Invitrogen, catalog #A11081) and AlexaFluor-488-conjugated goat anti-rabbit (Invitrogen, catalog #A11034) secondary antibodies for 2 h at room temperature. Nuclei were counterstained with DAPI (Fluka, 32670). Sections were mounted with anti-fading mounting medium Shandon Immuno Mount (Thermo Scientific, 9990402) on Super Frost Plus glass slides (R. Langenbrinck, 03-0060). Microphotographs were taken with a confocal microscope (Leica TCS SPE; RRID: SciRes_000154).

### Statistical analysis and methods to prevent bias

Power calculation was performed with R statmod-package v1.4.32 for Fisher’s exact test based on allelic association for the SVID cohort. The study had at least 80% power for the detection of common variants, MAF > 5%, with strong effect (OR < 0.6 or > 2), with a significance value of two-sided α = 0.05 (Fig. S1).

The joint effect of *Phactr1* coding and non-coding variants on vascular traits was performed with two Sample t-test in R (version × 64 3.0.2, http://www.r-project.org/).

Low frequency and rare variants were defined as having a 1% < MAF < 5% and MAF < 1%, respectively, either in cases or controls. Minor allele frequency was based either on HEX database for elderly controls > 70 years of age or ExAC database version 0.3.1 database (http://exac.broadinstitute.org/).

Mice were randomized to receive hypoperfusion.

### Bioinformatics, exome and genome sequencing

The reads were aligned using BWA-MEM v0.7.15 ^33^ to the reference GRCh37 (hs37d5.fa), separate read groups were assigned for all reads from one lane, and duplicates were masked using Samblaster v0.1.24^34^. Standard QC was performed using FastQC (http://www.bioinformatics.babraham.ac.uk/projects/fastqc). The variants were then called using GATK UnifiedGenotyper v3.7^35^ and annotated using Jannovar v0.24^36^ using RefSeq v105 exons.

For the CNV analysis of the WGS data, Cnvkit in batch mode was used in a matched fashion as described in their manual for WGS data.

### Angiotool

MCAO vessel length and total vascular junctions were calculated for leptomeningeal microvessels, selecting always the same regions of interest (both in terms of brain area in both hemispheres and region of interest dimension) by using the software AngioTool v 0.6a as previously described ^27^.

All methods were carried out in accordance with relevant guidelines and regulations.

## Results

### Exome sequencing in SVID-CADASIL-like patients

**Figure 3:**
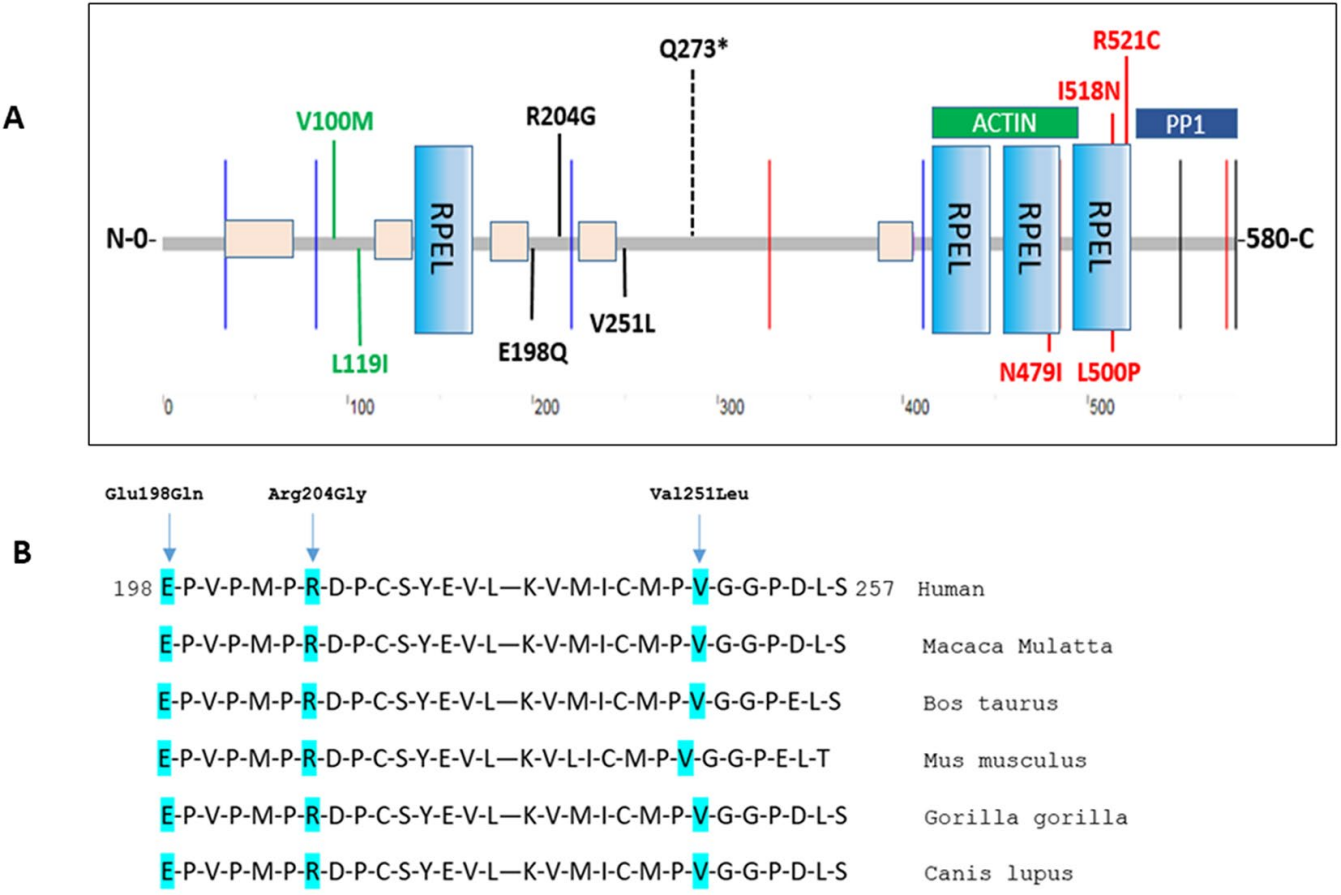
A. PHACTR1 protein structure. In black *PHACTR1* coding mutations detected in our SVD-CADASIL-like cohort and in the BCCAS-MCAO mouse cohort (dashed line) (Given the high level of analogy between the human and mouse PHACTR1 protein, we adopted the same human protein to show the coding variants detected both in patients and mice). In green *PHACTR1* coding mutations detected in elderly neuropathologically proven controls (478 controls > 60 years, HEX DATABASE). In red, *PHACTR1* coding pathogenic mutations in RPEL 3 and 4 domains, causative for West syndrome and other neurodevelopmental disorders ^11^. PHACTR1 protein structure has been adapted from String version 11.0 (https://string-db.org/). (**B**) PHACTR1 partial protein sequence, displaying a high degree of conservation across different species for Gly-198, Arg-204 and Val-251 amino-acids, where we detected *PHACTR1* coding mutations in our SVD-CADASIL-like cohort. (**C**, **D**) *Phactr1* in mouse brain. (**C**) Coronal mouse brain section showing *Phactr1* exclusive expression in neurons and to a significant lesser extent in the white matter (**D**). Scalebar A = 1000 μm and B = 200 μm.

In our SVID-CADASIL like cohort 3/180 patients (1.7%) carried three rare heterozygous coding variants in *PHACTR1* (p.Glu198Gln, p.Arg204Gly, p.Val251Leu) (Table 3). Among these, one was novel (p.Arg204Gly) and two were very rare coding variants (MAF < 1 × 10e − 5) (p.Glu198Gln, p.Val251Leu). These have been predicted likely pathogenic (CADD > 15), cluster in well conserved protein domains (Fig. 3) and the carrier frequency in our cohort was higher, although not significantly (Fisher’s exact test p-value < 0.05), when compared to 478 healthy neuropathologically proven controls (HEX database) (0.4%). However, these variants have been detected in exon 7 and 8 and, analogously to the *PHACTR1* variants found in controls, between 2 different low complexity regions (https://string-db.org/), outside the three RPEL regions or C terminal domain, actin and protein phosphatase 1 (PP1) binding sites, respectively, where all the *PHACTR1* pathogenic mutations have been reported in West syndrome and other neurodevelopmental disorders ^11,37^ (Fig. 3).

**Table 3:** *PHACTR1* coding variants detected in the SVID-CADASIL-like cohort and in the HEX database cohort of controls.

Patient ID	Gene	Position	rsID	Change	cDNA	Aa	ExAC	CADD	Exon	Domain	Phenotype	Origin	Gender	AAO	*APOE*	Lesions	Family history	Vascular risk factors	*NOTCH3*
Patient 1	*PHACTR1*	6:13182864	Novel	A/G	c.610A>G	p.Arg204Gly	NA	15.99	ex7	Low complexity	SVID	US	Male	52	ε3/ε2	SVID	Neg.	No	No
Patient 2	*PHACTR1*	6:13182846	rs376126977	G/C	c.592G>C	p.Glu198Gln	2.531e − 5	23.5	ex7	Unknown region	CADASIL-like	Portugal	Female	59	ε3/ε3	Vascular lesions		No	No
Patient 3	*PHACTR1*	6:13206133	rs375123444	G/T	c.751G>T	p.Val251Leu	9.949e − 5	15.30	ex8	Unknown region	CADASIL-like	Portugal	Male	57	ε4/ε4	Multi-lacunar infarcts	Neg.		No
CTRLS_HEX	*PHACTR1*	6:12933912	rs144313630	G/A	c.475 G>A	p.Val100Met	4.459e − 3	9.624	ex5	Unknown region	HEX controls		NA	> 60		No	NA	NA	NA
CTRLS_HEX	*PHACTR1*	6:12933969	rs78704568	C/A	c. 532C>A	p.Leu119Ile	1.608e − 3	4.038	ex5	Unknown region	HEX controls		NA	> 60		No	NA	NA	NA

*CTRLS* controls, *Aa* amino-acid, *AAO* age at onset, *SVID* small vessel ischemic disease, *Neg.* negative.

All the three *PHACTR1* rare variant mutation carriers, one from US with SVID and two from Portugal with CADASIL-like disease displayed an early-age at onset (average age at onset = 56 range 52–59) (Table 3). Moreover, none of the *PHACTR1* missense mutation carriers presented coronary artery disease or migraine, two traits already significantly associated to *PHACTR1* common non-coding variability ^1,2^. One carrier from the US was affected by hypertension, nonetheless, given the high frequency of hypertensive patients in the US cohort (60.4%), we may probably exclude a direct or critical effect of *PHACTR1* coding variability on hypertension.

### Genome sequencing and exome sequencing in BCCAS and MCAO mice characterized by different PcomA patency

#### *Phactr1* CNV in BCCAS

A growing body of evidence reported copy number variants (CNVs) as main mechanism of genome evolution ^38,39^. Considering that highly inbred strains like the C57BL/6J are not isogenic ^30,40^ and different vascular phenotypes such as the diverse degree of PcomA patency segregate within the strain, represent known phenotypic intrastrain differences ^19,27^ and may have been positively selected through the generations, we investigated the possibility that a CNV in *Phactr1* may have determined the different vascular phenotype in C57BL/6J BCCAS mice. We recently described the main arterial collateral compensatory mechanisms in BCCAS ^27^ and these primarily involve the PcomA patency. Therefore, we selected BCCAS mice with opposite collateral plasticity phenotype: (1) very effective collateral vascularization: two very prominent PcomAs leading to no lesion (defined as gray or white matter hyperintensities) detectable on T2 weighted MRI either 2 days or 7 days post-surgery (three mice) (Fig. 2-BI) and (2) ineffective vascular phenotype: absence of PcomA collateral flow, leading to severe ischemic hemispheric lesions and to the death of mice between a few hours and 1 day post-surgery (three mice) (Fig. 2-BII) (Table 2).

**Figure 4:**
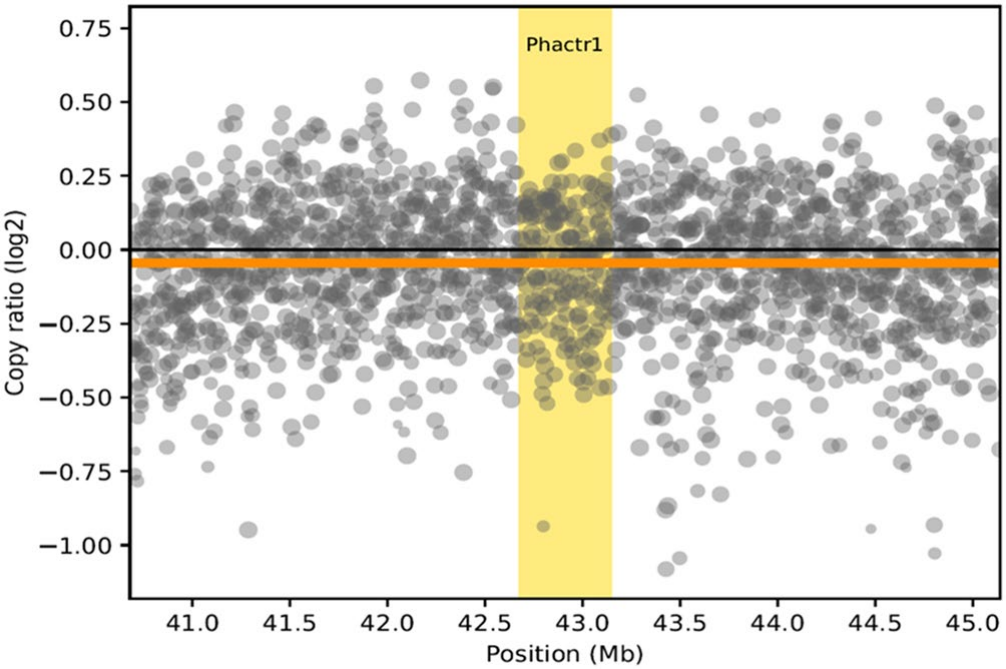
Copy number variant plot (CNV) plot across *Phactr1* locus based on genome sequencing data from BCCAS mice with different vascular phenotypes: three BCCAS mice with two very prominent PcomAs and ECA retrograde flow, which did not develop any ischemic lesion detectable on T2-weighted MRI both 1 and 7 days post-surgery (Fig. 2BI) and 3 BCCAS mice with no PcomAs and ECA retrograde flow,which developed severe hemispheric lesions and died within a few hours-one day post-surgery (Fig. 2BII).

We do not report any CNV in *Phactr1*, segregating with BCCAS with effective or not effective vascular traits (Fig. 4). Therefore, we may exclude that *Phactr1* CNV may influence PcomAs patency. We next focused on point mutations as another likely genetic mechanism explaining intrastrain vascular differences and included in the study another stroke experimental mouse model (MCAO) with C57BL/6J background, characterized by left hemispheric strokes, whose severity is directly proportional to the left PcomA size^19,27^.

We performed exome sequencing in nine MCAO and three BCCAS mice with diverse PcomA calibre and investigated the hypothesis that PcomA spectrum size, ranging from no PcomA/non-patent PcomA to very prominent PcomAs may have been determined by coding missense mutations in *Phactr1* (Fig. 2) (Table 4).

**Table 4:** *Phactr1* coding and non-coding variants detected in MCAO and BCCAS mice with exome sequencing.

Position	Type	cDNA	Aa	Effective vascular phenotype left prominent/very prominent PcomA			Effective vascular phenotype 2 prominent PcomAs ECA retrograde flow			Not effective vascular phenotype left non-patent PcomA			Not effective vascular phenotype left small PcomA		
				16_MCAO	17_MCAO	9_MCAO	BCCAS_13d	BCCAS_17d	BCCAS_24d	2_MCAO	4_MCAO	10_MCAO	12_MCAO	18_MCAO	1_MCAO
13:42709569	Intron	c.104-135T>C	–												
13:42709606	Intron	c.104-98T>C	–												
13:42709611	Intron	c.104-93T>C	–												
13:42868858	Intron	c.230-87759A>G	–												
13:42869103	Intron	c.230-87514G>T	–												
13:42957618	Intron	c.394 + 837C	–												
13:42958626	Intron	c.394 + 1845G>T	–												
13:42958828	Intron	c.394 + 2047G>T	–												
13:43059723	exon	c.817C>T	p.Gln273*												
13:43137028	3_prime_UTR	c.*1839C>A	–												
13:43137210	Intron	c.*2021C>A	–												
13:43137462	3_prime_UTR	c.*2273C>A	–												
13:43138151	3_prime_UTR	c.*2962C>A	–												
13:43138188	3_prime_UTR	c.*2999G>A	–												
13:43138227	3_prime_UTR	c.*3038C>A	–												

#### Single-variant study in BCCAS and MCAO mice

In the 12 MCAO-BCCAS exomes, we identified 15 variants in *Phactr1*: 14 non-coding variants and one heterozygous stop-gain mutation (p.Gln273*). The most common non-coding variant detected was c.104-93T>C, carried by 8/12 (66.66%) mice. The maximum number of variants carried pro mouse was four non-coding variants, found in a BCCAS mouse (BCCAS_17) with a very effective vascular phenotype and, given the lack of genetic variants in *Phactr1* in another BCCAS mouse (BCCAS_24) with prominent arterial collaterals, we may rule out a significant correlation between the enrichment for variants in *Phactr1* and a specific vascular phenotype. Most of the variants (85.7%) were singletons (Table 4). *PHACTR1* p.Gln273* clusters in exon 5, outside the REPL regions or C terminus, reported as *PHACTR1* functional domains^11,37^. *Phactr1* p.Gln273* has been detected in one MCAO mouse characterized by a left non-patent PcomA, which developed a severe left hemispheric stroke and died few hours after the surgery (Fig. S2). This variant did not segregate with this specific vascular trait, as it was not detected in the other MCAO mice with non-patent/small left PacomA (2/3 mice, respectively) (Table 4). Therefore, we may also rule out the possibility that coding mutations clustering outside Phactr1 functional domains may play a critical role in determining PcomAs recruitment or patency.

#### *Phactr1* pooled variants in BCCAS and MCAO mice

**Figure 5:**
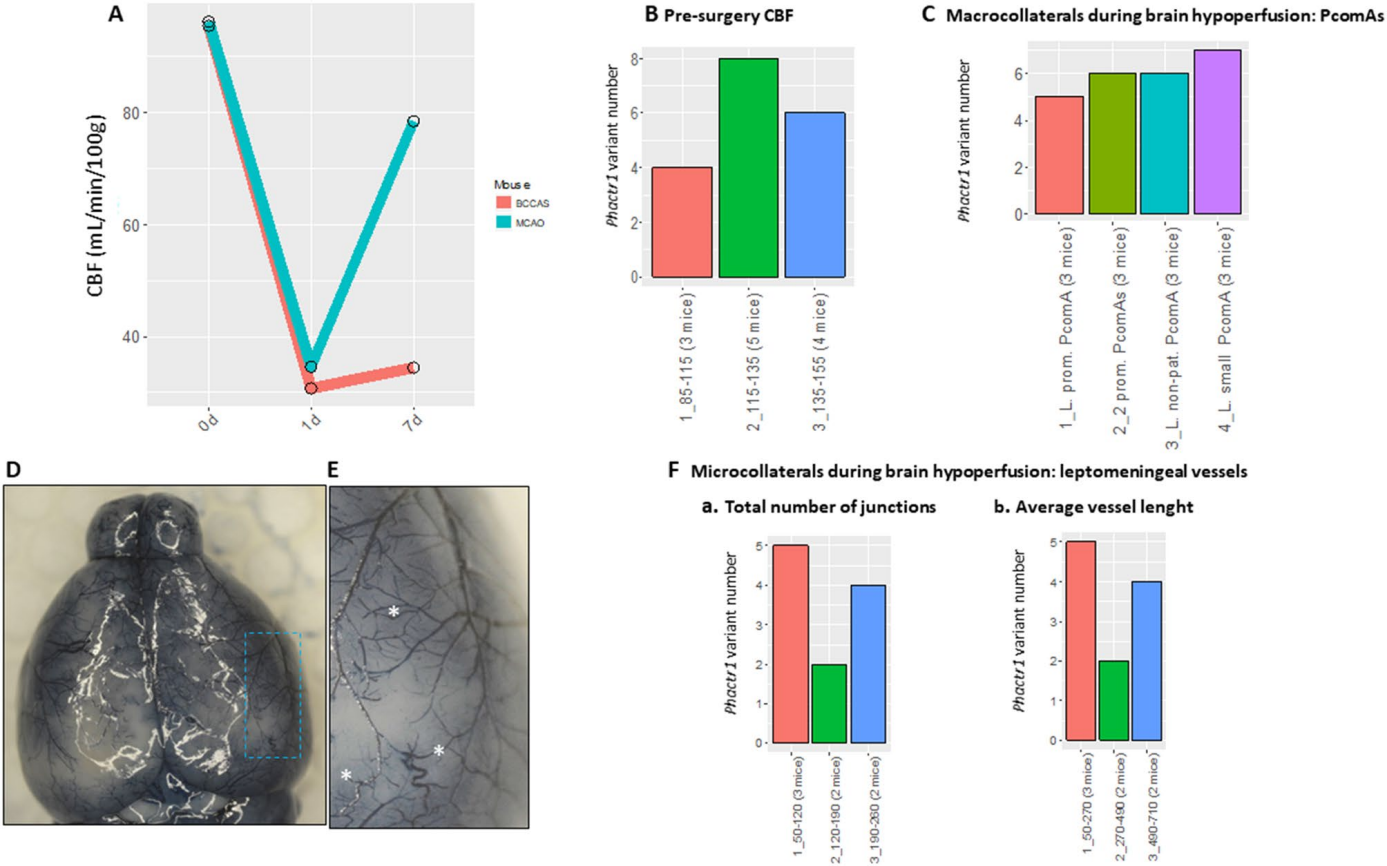
*Phactr1* pooled variant analysis in the BCCAS and MCAO mouse cohort. (**A**, **B**) Analysis of the cerebral blood flow (CBF) pre-surgery. (**A**) CBF pre-surgery and its severe drop (≈ 60–70% of the CBF pre-surgery) 1 day post-surgery and progressive recovery 7 day post-surgery, in BCCAS and MCAO mice. The graph was generated pooling CBF values for left striatum for 6 BCCAS and MCAO mice. (**B**) CBF values pre-surgery obtained pooling the CBF values measured in right and left cortex and striatum in MCAO (nine mice) and BCCAS (three mice). CBF values are expressed in ml/min/100 g. (**C**) Analysis of macrocollaterals: PcomA size. (**D**–**F**) Analysis of the leptomeningeal microcollaterals. (**D**) BCCAS mouse 7 days post-surgery, perfused with Evans Blue, axial view. (**E**) Anastomoses between leptomeningeal microcollaterals (asterisk). (**F**) Leptomeningeal vessels in the MCAO model (left hypoperfused hemisphere): a. average vessel length and b. total number of junctions. Some of these data have been already published ^27^.

We then investigated the hypothesis that the synergic effect of *Phactr1* coding and non-coding variants may have influenced the vascular phenotype in BCCAS-MCAO C57BL/6J mice. Particularly, we focused on a potential *Phactr1* variant enrichment as determinant for different vascular traits such as (1) embryonic vasculogenesis and vessel density indirectly measured through CBF in naive mice pre-surgery ^41^ (Fig. 5A,B, Table S1); (2) PcomA size during brain ischemia (Fig. 5C, Table 4) and (3) leptomeningeal microcollateral length and junction density during subacute hypoperfusion (7d) (Fig. 5D–F, Tables S2 and S3).

We do not report any significant association (t-test p-val < 0.05) between enrichment for *Phactr1* variability in mice and severity of different vascular traits at different time points both when considering CBF pre-surgery in naive mice or macro and microcollaterals (PcomA size and leptomeningel microvessel average length and number of junctions) during brain hypoperfusion (Fig. 5).

## Discussion

In this study we tested the hypothesis that genetic variability in *PHACTR1* may have significantly influenced the risk for ischemic stroke, either increasing the susceptibility to small vessel ischemic and CADASIL-like diseases in patients or regulating the recruitment and plasticity of PcomAs in 2 C57BL/6J experimental stroke mouse models (BCCAS and MCAO).

In the SVID-CADASIL-like disease cohort, we report three very rare coding variants (p.Glu198Gln, p.Arg204Gly, p.Val251Leu) clustering outside the PHACTR1 known functional domains, RPEL and C-terminus domains, harbouring the binding sites for g-actin and PP1, respectively. These domains have been reported to be critical for PHACTR1 regulation of angiogenesis ^42^ and to harbour causative mutations for West syndrome and other neurodevelopmental disorders ^11^. Mutations outside the RPEL and C-terminus domains have not been reported linked to any disorder or trait. Analogously to other proteins playing a key role in neurodegenerative disorders such as PSEN1, PSEN2, APP and CSF1R, whose pathogenic mutations cluster in definite functional domains (alpha-helix domain in the transmembrane domains, secretase domain and tyrosine kinase domains, respectively) ^43,44^ (www.molgendatabase), it is probable that only *PHACTR1* rare coding variants in the RPEL or C-terminus domains may manifest phenotypically.

Indeed, although *PHACTR1* p.Glu198Gln, p.Arg204Gly and p.Val251Leu have been reported as likely pathogenic (CADD > 15) and are well conserved across different species, rare coding mutations in PHACTR1 low complexity regions have been detected also in elderly neuropathologically proven controls (Fig. 3, Table 3).

Moreover *PHACTR1* p.Glu198Gln, p.Arg204Gly and p.Val251Leu carriers did not display migraine or coronary artery disease, both traits reported to be significantly associated to *PHACTR1*^1,2^.

Importantly, although we cannot exclude that rare coding variants in PHACTR1 functional domains may be causative or risk factors for ischemic stroke, cerebrovascular accidents have not been reported in West syndrome patients. However, given the rare incidence of the disorder (about one in 3000 children), the young age of the cases described (oldest patient reported = 28 years) ^45^ and the exclusive report of de novo mutations and not germline ones ^11^, we may not rule out that (1) these patients affected by epileptic encephalopathy with very early-onset may not develop ischemic strokes later in life, (2) undiagnosed lacunar ischemic lesions may have triggered epileptic seizures in these patients and (3) unlike germline mutations, not reported in PHACTR1 functional domains, de novo mutations may not significantly compromise pre-existent effective arterial compensatory mechanisms and therefore lead to a less severe phenotype without cerebrovascular episodes.

The lack of a significant correlation between *PHACTR1* genetic variability and ischemic stroke has been supported also by our experiments in brain ischemia mouse models (BCCAS and MCAO), where we do not report *Phactr1* CNVs as responsible for PcomA patency. Analogously, we found a *Phactr1* heterozygous stop-gain mutation (p.Gln273*), clustering outside the RPEL or C-terminus domains in one MCAO mouse with left non-patent PcomA, developing severe hemispheric strokes and dying few hours after surgery. Importantly, no coding mutations in *Phactr1* have been detected in other MCAO mice with similar vascular phenotype (left non-patent/small PcomA) and stroke outcome, suggesting that this variant does not segregate with a specific vascular trait and it is not likely to represent a pivotal factor determining PcomA patency.

We do not report any significant enrichment for *Phactr1* coding and non-coding variants linked to particularly effective or non-effective vascular traits (vascular density pre-surgery, indirectly detected through CBF pre-surgery, PcomA size, microcollateral leptomeningeal density and average vessel length during subacute hypoperfusion in mice) (Fig. 5). Thus, implying that also the joint effect of *Phactr1* variants may not result in a distinctive vascular phenotype.

The strength of our study lies in the use of inbred mouse genetics to interpret and complement the more complex genetic variability of independent familial and apparently sporadic patients. The selection of inbred mice with opposite vascular phenotypes (3–6 mice with either prominent/very prominent PcomA or small/non-patent PcomA), allowed us to reach, despite the small sample size, a relatively high power for the detection of rare coding functional variants. This enabled us to support the *PHACTR1* findings in the SVID and CADASIL-like underpowered patient cohort.

In summary, our study shows that *PHACTR1* rare coding variability outside the functional domains is unlikely to play a critical role in small vessel ischemic disease and CADASIL-like syndrome as well as brain collateral artery recruitment during brain hypoperfusion in mouse models. However, *PHACTR1* coding variability in RPEL domains are worth investigating in a larger cohort of SVID patients, in different ischemic stroke subtypes and with functional studies.

## Supplementary Information

Supplementary Information 1.

Supplementary Figure S1.

Supplementary Figure S2.

Supplementary Tables.

## Data Availability

All data generated or analysed during this study are included in this published article (and its “Supplementary Information” files).
