# Supplementary material for: PHACTR1 genetic variability is not critical in small vessel ischemic disease patients and PcomA recruitment in C57BL/6J mice
Source: Sci Rep. 2021 Mar 16;11:6072. doi: 10.1038/s41598-021-84919-x (PMC7966789; doi:10.1038/s41598-021-84919-x)
Supplement: Supplementary file 1 — Supplementary Information 1. [file 41598_2021_84919_MOESM1_ESM.docx]

*PHACTR1* genetic variability is not critical in small vessel ischemic disease patients and PcomA recruitment in C57BL/6J mice.

Clemens Messerschmidt^2^, Marco Foddis^1^, Sonja Blumenau^1^, Susanne Müller^1^, Kajetan Bentele^2^, Manuel Holtgrewe^2^, Celia Kun-Rodrigues^3^, Isabel Alonso^4^, Maria do Carmo Macario^5^, Ana Sofia Morgadinho^5^, Ana Graça Velon^6^, Gustavo Santo^5,8^, Isabel Santana^5,7,8^, Saana Mönkäre^9,10^, Liina Kuuluvainen^11,12^, Johanna Schleutker^10^, Minna Pöyhönen^9,11^, Liisa Myllykangas^13^, Assunta Senatore^14^, Daniel Berchtold^1^, Katarzyna Winek^1^, Andreas Meisel^1^, Aleksandra Pavlovic^15^, Vladimir Kostic^15^, Valerija Dobricic^15,16^, Ebba Lohmann^17,18,19^, Hasmet Hanagasi^17^, Gamze Guven^20^, Basar Bilgic^17^, Jose Bras^3^, Rita Guerreiro^3^, Dieter Beule^2^, Ulrich Dirnagl^1^, Celeste Sassi^1^

^1^Department of Experimental Neurology, Center for Stroke Research Berlin (CSB), Charité - Universitätsmedizin Berlin, Corporate Member of Freie Universität Berlin, Humboldt-Universität zu Berlin, and Berlin Institute of Health, Berlin, Germany

^2^Berlin Institute of Health, BIH, Core Unit Bioinformatics and Charité – Universitätsmedizin Berlin, Berlin Germany.

^3^Center for Neurodegenerative Science, Van Andel Research Institute, Grand Rapids, Michigan

^4^CGPP and UnIGENe, Instituto Biologia Molecular Celular, Instituto de Investigação e Inovação em Saúde, Porto, Portugal

^5^Department of Neurology, Centro Hospitalar e Universitário de Coimbra, Coimbra, Portugal

^6^ Department of Neurology, Centro Hospitalar Trás-os-Montes e Alto Douro, Portugal

^7^Faculdade de Medicina da Universidade de Coimbra, Coimbra, Portugal

^8^Centro de Neurociências e Biologia Celular da Universidade de Coimbra, Coimbra, Portugal

^9^Department of Medical Genetics, University of Helsinki, Helsinki, Finland

^10^Turku University Hospital, Laboratory Division, Genomics, Department of Medical Genetics, Turku, Finland

^11^Department of Clinical Genetics, Helsinki University Hospital

^12^Department of Medical Genetics, University of Helsinki, Helsinki, Finland

^13^Department of Pathology, University of Helsinki and Helsinki University Hospital, Helsinki, Finland.

^14^Institute of Neuropathology, University of Zurich, Zurich, Switzerland.

^15^Clinic of Neurology, Faculty of Medicine, University of Belgrade, Belgrade, Serbia.

^16^Lübeck Interdisciplinary Platform for Genome Analytics (LIGA), Institutes of Neurogenetics & Cardiogenetics, University of Lübeck, Lübeck, Germany.

^17^Behavioural Neurology and Movement Disorders Unit, Department of Neurology, Istanbul Faculty of Medicine, Istanbul University, Istanbul, Turkey

^18^Department of Neurodegenerative Diseases, Hertie Institute for Clinical Brain Research, University of Tübingen, Tübingen, Germany

^19^DZNE, German Center for Neurodegenerative Diseases, Tübingen, Germany

^20^Department of Genetics, Aziz Sancar Institute of Experimental Medicine, Istanbul University, Istanbul, Turkey

**Corresponding author:**

Celeste Sassi, MD, PhD

Klinik und Poliklinik für Neurologie , Abteilung für Experimentelle Neurologie, Charité – Universitätsmedizin Berlin

Charitéplatz 1, D-10117 Berlin

Tel.: 030 – 450 560 149, Fax: 030 – 450 560 915, [celeste.sassi.10@alumni.ucl.ac.uk](mailto:celeste.sassi.10@alumni.ucl.ac.uk)

**SUPPLEMENTARY MATERIALS AND METHODS**

**BCCAS**

Anaesthesia was achieved using isoflurane in a 70:30 nitrous oxide:oxygen mixture and core body temperature was maintained at 37 ± 0.2 °C with an automated rectal probe and heat blanket. A midline incision was made in the neck, and a carotid artery was carefully exposed. Hypoperfusion was induced by winding a custom ordered, non-magnetic, surgical grade microcoil (160 µm inner diameter, Shannon Coiled Springs Microcoil, Limerick, Ireland) around one of the carotid arteries. The sham procedure was performed with a larger diameter microcoil (500 µm) that did not constrict the vessel. The muscle and glands were guided back into place and local anaesthetic was applied to the sutured wound prior to recovery. Twenty- four hours later, the same procedure was repeated on the other carotid artery. This delay represents an important refinement that does not result in higher mortality when using the smaller sized microcoils. Regular diet was placed on the floor of the cage to assist with feeding, and animals were provided with 6 mg/mL of Paracetamol in the drinking water to assist with post-operative pain (one day prior to, and up to three days post-surgery).

**MCAO**

In the MCAO model, hypoperfusion was induced as described previously (http://precedings.nature.com/documents/3492/version/2). Briefly, after closing the left common carotid artery (CCA) and left external carotid artery (ECA) a microvascular clip was put on the left internal carotid artery (ICA) and a small incision was made on the CCA. A nylon filament (7019PK5Re, Doccol Corp, Redlands, California, USA) was introduced over CCA and ICA to occlude the origin of middle cerebral artery (MCA) and fixed with a suture around the ICA. After 60 minutes, the filament was removed causing immediate reperfusion. Afterwards the suture on the ICA was closed. This type of MCAO surgery results in permanent occlusion of left CCA, ECA and ICA.

**MRI measurements**

Anaesthesia was again achieved using isoflurane as per above, and body temperature and respiration rate were monitored with MRI compatible equipment (Small Animal Instruments, Inc., Stony Brook, NY).

*Cerebral blood flow and angiographies*

CBF and angiography were measured on a 7 T Pharmascan using Paravision 5.1 software (Bruker BioSpin, Ettlingen, Germany). For the CBF measurement, radio frequency transmission was achieved with a 72 mm diameter quadrature resonator actively decoupled to a mouse quadrature surface coil used for reception (Bruker BioSpin, Ettlingen, Germany). A single slice (1 mm) flow-sensitive alternating inversion recovery (FAIR) sequence with a rapid acquisition with relaxation enhancement (RARE) readout was used (repetition time (TR)/recovery time/echo spacing (ΔTE)/effective echo time (TEeff): 12 000/10 000/7.2/35.9 ms, respectively, 16 inversion times (35-1500 ms), RARE factor: 32, inversion slice thickness: 4 mm, 180° hyperbolic secant (sech80) inversion pulse (20 ms), field of view (FOV): 25.6 mm2, matrix: 128 x 64 enlarged by partial fourier transform to 128 x 128, resolution: 200 µm2, 12 min). For angiography measurements, a 20 mm diameter quadrature volume coil (RAPID Biomedical, Rimpar, Germany) was used for radio frequency transmission and reception and a 3D time of light (TOF) sequence was used (TR/TE: 15/2.5 ms, α: 20 ˚, FOV: 25 mm3, resolution: 98 x 130 x 196 µm3 zero-filled to 98 µm3, 6 min). Spectroscopy, T2 weighted and MR spectra were acquired on a 7 T Biospec with a cryogenically cooled transmit/receive surface coil and Paravision 6.0 software (Bruker BioSpin, Ettlingen, Germany).

A 2D RARE T2 sequence was used for anatomical images (TR/ΔTE/TEeff: 3100/11/33 ms, RARE factor: 8, 29 consecutive slices, slice thickness 0.45 mm, FOV: (16.2 mm)2, resolution: 100 µm2, NA: 2, 2 min 4 s). A stimulated echo acquisition mode (STEAM) sequence was used for spectroscopy following local shimming (MAPSHIM) across a cubic 8 mm3 voxel placed in the striatum (TR/TE/mixing time: 2500 ms/3 ms/10 ms, number of averages (NA): 256, VAPOR water suppression, 10 min 40 s).

**MRI Data Analysis**

CBF maps were calculated using the Perfusion ASL macro in Paravision 5.1 software via the T1 method using a blood T1 value of 2100 ms and a brain blood partition coefficient of 0.89 mL/g ^1, 2^. Analysis of the CBF values were done using a custom written Matlab toolbox for nonlinear atlas registration ^3^ was used to select the CBF slice from the volume and coregister the CBF on the T2 images. Finally T2 and CBF images were transformed into the Allen brain atlas space and the atlas based CBF-values were extracted for all correlating Allen brain atlas structures in both hemispheres. (Release 2013a (MathWorks, Natick, MA, USA) script extracted the CBF maps from Paravision, and used atlas registration and coregistration of CBF maps in the atlas space for striatum and prefrontal cortex). The resulting CBF values were expressed in mL/min/100g.

**Tissue preparation and staining procedures (does not apply to BCCAS mice used for RNAsequencing)**

At the conclusion of the experiments, mice were deeply anaesthetized with ketamine and xylazine and perfused through the heart with physiological saline followed by 4% paraformaldehyde, Alexa Fluor 680 conjugate of WGA, Termofisher, W32465, 3% Gelatin (Sigma-Aldrich, [G1890](https://www.sigmaaldrich.com/ProductLookup.html?ProdNo=G1890&Brand=SIGMA)), 1% low melting agarose (Sigma Aldrich A4018) and 0.1% Evans Blue (Sigma Aldrich E2129). Whole brains were scanned with Li-cor (Li-Cor Odyssey-CLx). Subsequently, the brains were post-fixed for 24 hours in 4% PFA, and cryoprotected in 30% sucrose solution before being snap frozen in -40 ºC methylbutane. Tissue was sectioned to 50 µm and stored in cryo-protective solution (1 part ethylene glycol, 1 part glycerine and 2 parts phosphate buffered saline (PBS)) at -20 ºC.

**References**

1. Leithner, C. *et al.* Determination of the brain-blood partition coefficient for water in mice using MRI. *J. Cereb. Blood Flow Metab.* **30**, 1821–1824 (2010).

2. Dobre, M. C., Uğurbil, K. & Marjanska, M. Determination of blood longitudinal relaxation time (T1) at high magnetic field strengths. *Magn Reson Imaging* **25**, 733–735 (2007).

3. Koch, S. *et al.* Atlas registration for edema-corrected MRI lesion volume in mouse stroke models. *J. Cereb. Blood Flow Metab.* 271678X17726635 (2017) doi:10.1177/0271678X17726635.

**Figure S1**

Power calculation in our SVD cohort, showing that the study had 80% power to detect common variants with strong effect size. R statmod-package [v1.4.32](https://www.rdocumentation.org/packages/statmod/versions/1.4.32) for Fisher´s exact test based on allelic association was used for this power calculation. OR, odds ratio.

**Figure S2**

Phenotype of the MCAO mouse carrying *Phactr1* LoF (p.Q273*). **A**. Left hemispheric severe stroke with edema, axial view. **B**. Left severe stroke with edema and non-patent left PcomA (dashed lines). **C**. Coronal histological section stained with Gfap, displaying tissue edema and necrosis in the left cortex, corpus callosum and striatum and astrocytosis particularly in the corpus callosum. *Phactr1* in mouse brain (**D-E**). **D**. Coronal mouse brain section stained with PHACTR1 antibody and showing Phactr1 exclusive expression in neurons and to a significant lesser extent in the white matter (**D-E**). Scale bar A= 1000um and B =200um

**Table S1**

Cerebral blood flow pre-surgery (day 0) detected in striatum and cortex with arterial spin labelling in MCAO and BCCAS mice. In the study we used the mean value between left and right striatum and cortex. CBF values are expressed in mL/min/100g. Some of these data have been already reported ^4^

**Table S2**

Average leptomeningeal microvessel length, detected in the MCAO mice left hemisphere during hypoperfusion. Data are expressed in μm. Some of these data have been already reported ^4^

**Table S3**

Total number of leptomeningeal microvessel junctions detected in the MCAO mice left hemisphere during hypoperfusion. Some of these data have been already reported ^4^
