# Supplementary figures and images for: PHACTR1 genetic variability is not critical in small vessel ischemic disease patients and PcomA recruitment in C57BL/6J mice
Source: Sci Rep. 2021 Mar 16;11:6072. doi: 10.1038/s41598-021-84919-x (PMC7966789; doi:10.1038/s41598-021-84919-x)

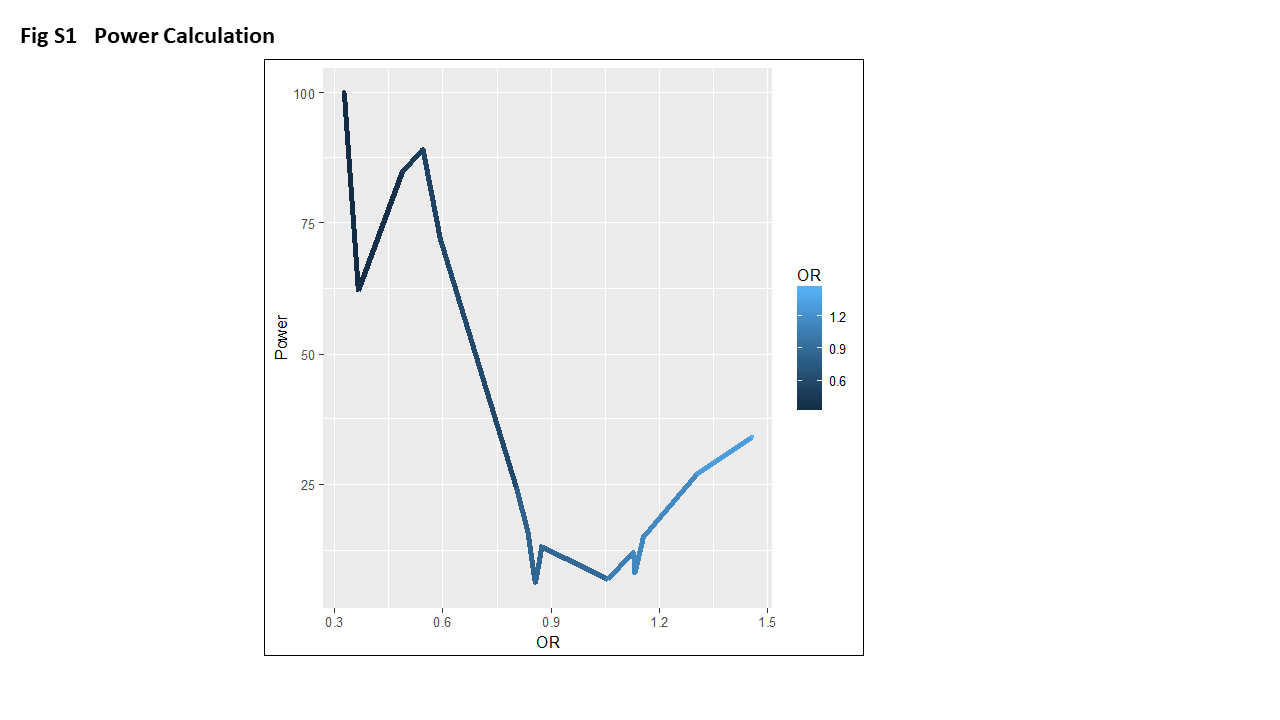

Supplement: Supplementary file 2 — Supplementary Figure S1. [file 41598_2021_84919_MOESM2_ESM.tif]

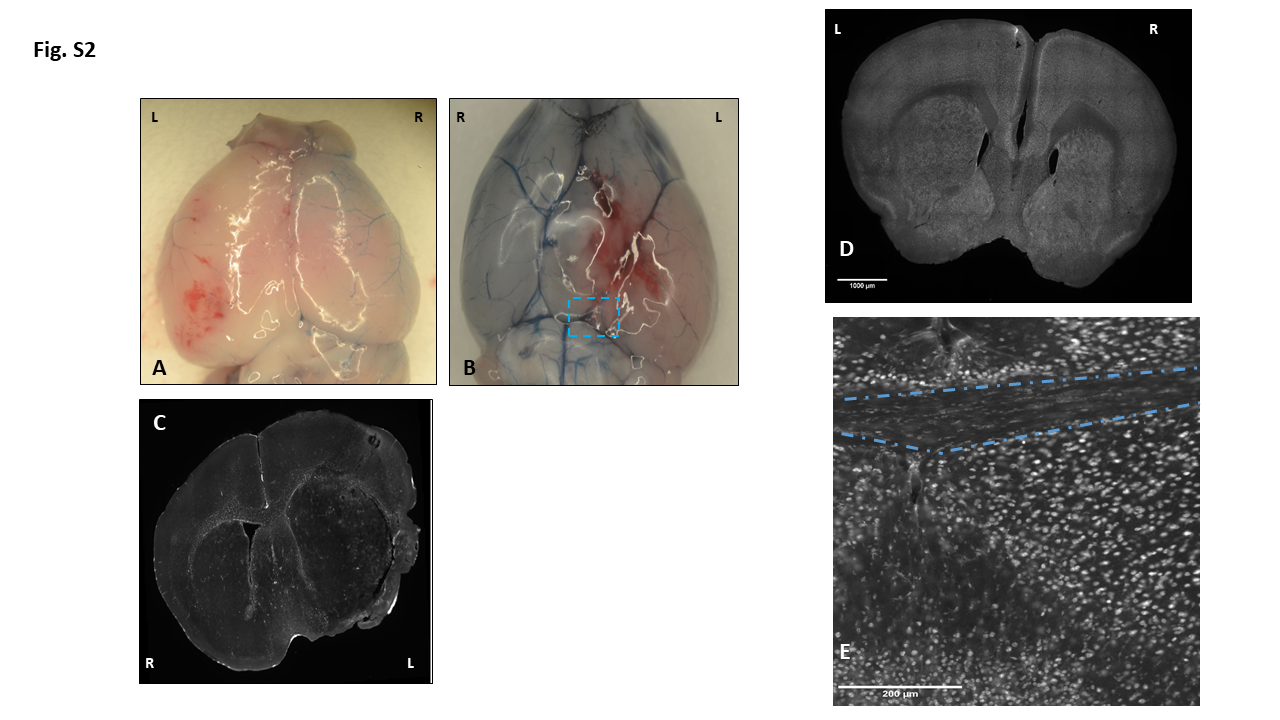

Supplement: Supplementary file 3 — Supplementary Figure S2. [file 41598_2021_84919_MOESM3_ESM.tif]
